# Psychosocial impact of melasma: Asian Americansand people with pre-existing mood or anxiety disorders are disproportionately affected

**DOI:** 10.21203/rs.3.rs-6382566/v1

**Published:** 2025-09-05

**Authors:** Brad R. Woodie, Muayad M. Shahin, Gabrielle M. Rivin, Heather C. W. Harrison, Alan B. Fleischer

**Affiliations:** College of Medicine, University of Cincinnati College of Medicine; College of Medicine, University of Cincinnati College of Medicine; Department of Dermatology, University of Cincinnati College of Medicine; School of Professional Psychology, Spalding University; Department of Dermatology, University of Cincinnati College of Medicine

**Keywords:** Hyperpigmentation, Quality of Life, Mental Health, Depression, Anxiety Disorders, Socioeconomic Factors, Race, Ethnicity, Case-Control Studies, Psychosocial Factors, Patient-Reported Outcomes

## Abstract

Melasma is a chronic hyperpigmentation disorder that primarily affects sun-exposed areas of the skin, especially the face. It is most common in adult women with darker skin phototypes (Fitzpatrick III and IV), and it is notoriously difficult to treat. As a result, melasma can adversely impact mental health and quality of life, with affected individuals having higher rates of depression. This study explored the psychosocial associations of melasma using data from the National Institutes of Health’s *All* of Us Research Program. A total of 746 participants with a melasma diagnosis who completed the “Overall Health” survey were identified and matched with control participants on age, sex, race, and income. Covariates such as duration of melasma, sunlight intensity, treatment history, and pre-existing psychiatric conditions were accounted for in multivariable logistic regression analyses within melasma and between cases and controls. Results revealed that self-reported Asian Americans with melasma had lower mental and social health scores compared with both non-Hispanic White Americans with melasma and Asian Americans without melasma. This association may partly result from beauty ideals in Asian American communities, where lighter skin is regarded as a symbol of privilege. Additionally, individuals with melasma and a pre-existing mood or anxiety disorder reported poorer mental health than those with only the psychiatric disorder. These findings suggest that the psychosocial impact of melasma may be magnified in Asian Americans and people with mood or anxiety disorders. This study emphasizes the need for psychological support for patients at higher risk of adverse mental health outcomes.

## Introduction

Melasma is a chronic hyperpigmentation disorder primarily affecting sun-exposed areas of the body, particularly the face. It is most common in adult women with darker skin phototypes (Fitzpatrick III, IV), and is notoriously difficult to treat, with a high recurrence rate [7, 19]. Due to its frequent involvement of the face and the challenges in achieving effective treatment, melasma significantly impacts patients’ quality of life. Mental health conditions, including anxiety, depression, and body dysmorphic disorder, are more common in people with melasma [3, 5, 8, 24]. Prevalence of these conditions varies by region, with patients with melasma in Asia showing the highest prevalence of depression [3].

Importantly, formal diagnosis of these comorbidities can be biased by factors such as access to healthcare, socioeconomic status, and individual health utilization behaviors. Diagnostic codes do not capture the subjective patient experience, which is important for understanding the overall psychosocial health burden in this population. Also, studies that have used diagnostic codes to identify psychiatric comorbidities in melasma patients often do not account for the temporal relationship between melasma and these mental health conditions. Further, patient characteristics that predict greater psychosocial impairment have not been comprehensively evaluated to our knowledge.

In this case-control study, we aim to explore the psychosocial impact of melasma, focusing on associations with race and ethnicity, mental health disorders, and socioeconomic status in the United States. The large and diverse *All of Us* research database, previously used to examine melasma risk factors [8], was utilized to investigate associations between melasma and psychosocial survey responses.

## Methods

We analyzed participant data from the National Institutes of Health’s *All of Us* Research Program, controlled tier version seven, which contains deidentified electronic health records of approximately 290,000 individuals. We identified 746 participants with a melasma diagnosis (Systematized Nomenclature of Medicine Clinical Terms [SNOMED] code 36209000) in their electronic medical record who also completed the “Overall Health” survey questions.

### Covariates and outcomes

Covariates included patient age, duration of melasma, sunlight intensity (mean annual global horizontal irradiance; GHI) of the participant’s postal code (obtained from the National Renewable Energy Laboratory [18]), sex assigned at birth, self-reported race, self-reported ethnicity, patient self-reported income, history of depression, history of hydroquinone use (including combination preparations), history of other common topical medications for melasma (azelaic acid, topical retinoids, niacinamide, tranexamic acid), prior use of combined oral contraceptives, past pregnancy, and past radiation (Online Resource 1). For participants who declined to report their income, the median household income from the participant’s postal code was used.

We also accounted for the five most common psychiatric diagnostic categories in the *All of Us* cohort (as determined by Barr. et al [2]), which included mood disorders (major depressive disorder, bipolar disorder, persistent depressive disorder (dysthymia)), anxiety disorders (unspecified anxiety disorder, generalized anxiety disorder, agoraphobia), substance use disorders (alcohol use disorder, tobacco use disorder, drug use disorders), stress-related disorders (post-traumatic stress disorder, adjustment disorder), and sleep disorders (insomnia, other specified sleep disorders) (Online Resource 2). To account for the possible role of pre-existing psychiatric disorders in psychosocial survey responses, the month of the first diagnosis of a mood disorder, anxiety disorder, etc. was compared with the month of the first diagnosis of melasma.

The *All of Us* program uses the Patient-Reported Outcomes Measurement Information System (PROMIS) Global Health survey to assess overall health [20]. Selected questions assessed mental, physical, and social health, and scoring of responses is shown in Table 1. Physical health was included to verify that participants were not experiencing difficulties due to other medical conditions unrelated to melasma. Survey responses of “poor” or “fair” were categorized as “low,” and responses of “good”, “very good”, or “excellent” were categorized as “high” as others have done previously [4,23].

Survey responses were first compared within participants with melasma with the above covariates and the outcome as low versus high response for each of the four survey questions.

### Case-control analyses

We then performed three case-control analyses to assess for independent contributions of race/ethnicity, mental health disorders, and income to psychosocial survey responses in participants with melasma. First, we propensity-matched participants with melasma (n=746) in a 1:10 ratio to participants without melasma (n=7,460) on age, sex, and income and compared survey responses, stratified by race and ethnicity. To determine if individuals with both melasma and a pre-existing mental health disorder report lower psychosocial health than individuals with only a mental health disorder, participants with melasma and each of the five categories of mental health disorders were propensity-matched in a 1:10 ratio on age, sex, race/ethnicity, and income to participants without melasma and the corresponding category of mental health disorder. Finally, to evaluate the possible role of income, we propensity-matched participants with melasma to participants without melasma according to age, sex, and race/ethnicity and compared survey responses, stratified by income.

### Statistical analysis

Multivariable logistic regression was performed for patients with melasma adjusting for the above covariates and the outcomes as low mental health, low ability to complete social roles, low social satisfaction, and low physical health. Multivariable logistic regression was then conducted for the propensity-matched cases and controls using the above covariates, with the exception of hydroquinone use for melasma, other topical use for melasma, and duration of melasma. Significance was set at two-tailed p<.05, and all analysis was performed in the SAS Studio environment within the *All of Us* Researcher Workbench.

## Results

Characteristics of the sample are shown in Online Resource 3 and distribution of responses is shown in Online Resource 4.

Adjusted for pre-existing and subsequent psychiatric conditions, self-reported Asian race predicted lower scores for mental and social health compared to White non-Hispanic participants (Table 2 and Online Resource 5). There were no significant differences in self-reported mental, social, or physical health between Black, Hispanic, or other race participants and White non-Hispanic participants. Lower income categories also predicted lower scores for mental, social, and physical health. Longer duration of melasma and greater sunlight intensity did not predict low psychosocial health.

Analysis of the matched cases and controls between race and ethnicity categories supported that Asian Americans with melasma report lower psychosocial health compared with Asian Americans without melasma (Table 3). There was no significant difference in survey responses between people with and without melasma within self-reported White race, Black race, other races, and Hispanic or Latino ethnicity.

Participants with melasma and a pre-existing mood disorder or melasma and a pre-existing anxiety disorder reported lower mental health than participants with only a mood disorder or anxiety disorder (Table 4).

Participants with melasma versus without melasma did not show significant differences in psychosocial health within income categories, with the exception of improved ability to perform social roles within the over $200k income group (Table 5).

## Discussion

Our findings indicate that self-reported Asian Americans with melasma experience worse mental and social health compared to self-reported White non-Hispanic Americans with melasma. Additionally, our case-control analysis shows that Asian Americans with melasma had lower psychosocial health scores when compared to Asian Americans without melasma.

This heightened psychosocial burden may stem from multiple sociocultural factors. One contributor may be beauty standards within Asian American communities, where lighter skin is often associated with privilege- a phenomenon linked to colorism [10]. In East Asian cultures, sun protection is frequently driven by the desire to prevent skin darkening, reflecting a traditional preference for fair skin [22]. As a result, darker skin can be seen as undesirable, perpetuating these cultural ideals. Tran et al. found that darker skin color in Asian American college students was correlated with higher rates of perceived prejudice and depression [21]. Since melasma leads to darkened skin and irregular pigmentation, it may be particularly distressing for this population, potentially explaining the heightened psychosocial stress among Asian Americans.

Furthermore, the widespread use of skin-lightening products in Southeast Asia [15] may also offer insight into why melasma has a stronger psychosocial effect on Asian Americans. While skin care practices in Asia are not identical to those in the United States, these cultural trends may help explain the greater psychological burden melasma places on Asian American individuals.

Another potential contributor to the psychosocial status of Asian Americans is perceived discrimination, particularly in healthcare settings. Asian Americans are disproportionately affected by discrimination in healthcare settings, and these experiences are associated with poor health outcomes [11–13]. Melasma, being a cosmetically sensitive condition, could increase perceived interpersonal discrimination in Asian Americans seeking care. Participants of other self-races and ethnicities who typically have skin of color (Black, Hispanic) did not show associations with psychosocial difficulty in the current study, possibly supporting the role of cultural norms in the psychological impact of melasma.

Melasma appears to have an additional impact in individuals with pre-existing mood or anxiety disorders. Perceived discrimination is also strongly associated with psychiatric disorders [1, 17], and people with melasma could have a heightened sensitivity to perceived cosmetic flaws. A study of people who underwent elective septorhinoplasty found that people with body dysmorphic disorder, anxiety, or depression experienced significantly less improvement in quality of life, suggesting that those with mood and anxiety disorders are more prone to worsened mental health as a result of perceived cosmetic imperfections [9]. Future research may be able to elucidate connections between melasma with specific neuropsychiatric diagnoses such as obsessive-compulsive disorder, which is independently associated with experienced stigma and embarrassment [6, 14]. Ultimately, clinicians should be aware that individuals with melasma and a pre-existing mood or anxiety disorder represent a higher-risk group for psychological complications of their skin condition and may benefit from additional psychological support.

Finally, while lower-income participants with melasma reported worse mental, social, and physical health, there was no significant difference when comparing them to similarly socioeconomically situated individuals without melasma. This suggests that the poorer psychosocial health observed in people with lower incomes (which is a known phenomenon^2^) is independent of melasma. Although a financial burden is not implied by our results, hydroquinone is available by prescription only in the United States and is generally not covered by insurance^16^ due to the “cosmetic” nature of the condition.

This study has limitations. Race and ethnicity are self-reported as social categories; thus, participant skin phototype is not known. Also, substantial cultural heterogeneity is present within races and ethnicities; the classifications in this study are a simplification and they cannot represent the experience of each included individual. Although the *All of Us* participant base includes individuals from diverse racial, ethnic, gender, and sexual backgrounds, it is not a representative sample of the United States. In addition, no established minimal clinically important difference exists for the surveys used in this study. A further limitation is the absence of a visual severity assessment. However, even when evaluated, objective measures of disease severity may not fully capture the patient’s subjective experience. Future studies could address these limitations by incorporating both objective ratings and patient-reported outcomes for melasma.

## Conclusion

Our study investigated the mental health impact of melasma on different groups in the United States, adjusting for pre-existing and subsequent diagnoses of psychiatric conditions. Asian Americans with melasma reported worse mental and social health compared to non-Hispanic White Americans with melasma and compared to Asian Americans without melasma. People with melasma and a pre-existing mood or anxiety disorder also report worse mental health than people with mood or anxiety disorders alone, suggesting that melasma may have an additional impact in people with these psychiatric diagnoses.

## Supplementary Files

This is a list of supplementary files associated with this preprint. Click to download.

- MelasmaOnlineResources.docx

## Figures and Tables

**Table 1:** Survey questions, responses, and binary categorization in the study.

Question	Response	Category
**In general, how would you rate your mental health, including your mood and your ability to think?**	Poor	Low
	Fair	
	Good	High
	Very good	
	Excellent	
**In general, please rate how well you carry out your usual social roles (This includes activities at home, at work and in your community, and responsibilities as a parent, child, spouse, employee, friend, etc.).**	Poor	Low
	Fair	
	Good	High
	Very good	
	Excellent	
**In general, how would you rate your satisfaction with your social activities and relationships?**	Poor	Low
	Fair	
	Good	High
	Very good	
	Excellent	
**In general, how would you rate your physical health?**	Poor	Low
	Fair	
	Good	High
	Very good	
	Excellent	

**Table 2:** Association of race and income with low mental and social health in n=746 participants with melasma, adjusting for pre-existing psychiatric conditions.

Comparison^a^	Low Mental Health	Low Social Roles	Low Social Satisfaction	Low Physical Health
Age (continuous)	1.0 (1.0, 1.0)	1.0 (1.0, 1.0)	1.0 (1.0, 1.0)	1.0 (1.0, 1.0)
Female vs Male	2.7 (0.6, 13)	2.3 (0.5, 11)	0.9 (0.3, 2.4)	2.9 (0.9, 9.3)
Other sex vs Male	3.7 (0.5, 28)	1.8 (0.2, 15)	1.5 (0.3, 6.8)	**13.6 (2.6–70)** **
Asian^b^ vs White	**4.9 (1.9, 13)** **	**3.5 (1.4, 9.2)** ***	**4.7 (2.0, 11)** ***	1.2 (0.4, 3.2)
Black vs White	1.2 (0.5, 2.8)	1.2 (0.5, 2.8)	1.5 (0.7, 3.2)	0.7 (0.4, 1.5)
Other race vs White	1.5 (0.6, 3.9)	1.1 (0.4, 3.1)	1.5 (0.6, 3.6)	0.7 (0.3, 1.6)
Hispanic vs White Non-Hispanic	**1.9 (1.1, 3.6)** *	1.5 (0.8, 2.9)	1.4 (0.8, 2.4)	**2.1 (1.3, 3.5)** **
<$25k vs Over 200k	**3.6 (1.2, 11)** *	**3.8 (1.1, 13)** *	**3.7 (1.3, 10)** *	**2.6 (1.1, 6.3)** *
$25k–50k vs Over 200k	2.2 (0.7, 6.8)	1.8 (0.5, 6.1)	2.0 (0.7, 5.6)	**2.5 (1.0, 5.9)** *
$50k–100k vs Over 200k	1.7 (0.6, 4.9)	2.2 (0.7, 6.7)	1.8 (0.7, 4.6)	1.2 (0.5, 2.7)
$100k–200k vs Over 200k	1.1 (0.4, 3.7)	1.3 (0.4, 4.6)	1.2 (0.4, 3.4)	0.5 (0.2, 1.4)
Pre-existing^c^ mood disorder	**2.8 (1.6, 5.0)** ***	**2.3 (1.3, 4.3)** **	**3.0 (1.7, 5.2)** ***	**2.7 (1.6, 4.3)** ***
Subsequent mood disorder	2.0 (0.9, 4.3)	1.9 (0.9, 4.1)	**2.5 (1.2, 5.2)** *	1.1 (0.6, 2.2)
Pre-existing anxiety disorder	**2.2 (1.2, 3.9)** *	1.2 (0.7, 2.2)	1.5 (0.9, 2.5)	1.1 (0.7, 1.9)
Subsequent anxiety disorder	**2.1 (1.1, 4.1)** *	1.5 (0.7, 2.9)	1.4 (0.7, 2.7)	1.0 (0.6, 1.9)
Pre-existing substance use disorder	1.3 (0.7, 2.7)	1.3 (0.6, 2.6)	1.5 (0.8, 2.9)	**2.4 (1.3, 4.6)** *
Subsequent substance use disorder	2.1 (0.4, 12)	1.9 (0.3, 11)	0.5 (0.1, 4.3)	2.6 (0.6,12)
Pre-existing stress disorder	1.1 (0.6, 2.0)	1.6 (0.9, 2.9)	1.3 (0.7, 2.3)	1.6 (1.0, 2.7)
Subsequent stress disorder	**2.7 (1.2, 5.7)** *	**2.7 (1.2, 5.8)** *	2.1 (1.0, 4.5)	1.6 (0.7, 3.4)
Pre-existing sleep disorder	1.2 (0.7, 2.2)	1.6 (0.9, 2.8)	1.1 (0.6, 1.8)	**1.7 (l.0–2.7)** *
Subsequent sleep disorder	0.9 (0.4, 1.8)	1.6 (0.8, 3.3)	1.3 (0.7, 2.4)	1.6 (0.9, 2.9)
Hydroquinone used	1.0 (0.6, 1.7)	0.8 (0.5, 1.3)	1.0 (0.6, 1.5)	0.9 (0.6, 1.5)
Other topicals used	1.1 (0.7, 1.8)	1.2 (0.7, 2.0)	0.9 (0.6, 1.5)	0.7 (0.4, 1.1)
Past pregnancy	1.2 (0.7, 2.0)	0.7 (0.4, 1.2)	**0.6 (0.3, 1.0)** *	0.7 (0.4, 1.1)
Past combined oral contraceptives	0.8 (0.5, 1.3)	0.9 (0.5, 1.5)	0.9 (0.5, 1.4)	0.8 (0.5, 1.3)
Past radiation	1.3 (0.7, 2.6)	1.1 (0.6, 2.1)	1.1 (0.6, 2.1)	0.8 (0.4, 1.5)
Global Horizontal Irradiance (continuous)	0.8 (0.5, 1.2)	1.0 (0.6, 1.5)	1.0 (0.6, 1.4)	0.8 (0.5, 1.1)
Duration (continuous)	1.0 (0.9, 1.1)	1.0 (0.9, 1.1)	1.0 (0.9, 1.1)	1.0 (0.9, 1.1)

a Adjusted odds ratios and 95% confidence intervals are shown.

* p<.05;

** p<.01;

*** p<.001.

b Race and ethnicity were obtained from self-report and were included in this analysis as a goal of *All of Us* to collect and report data from a diverse group of participants. “Other” races included American Indian or Alaskan Native, Middle Eastern or North African, Native Hawaiian or Other Pacific Islander, race not specified, and more than one population.

c “Pre-existing” and “Subsequent” refer to the date of first melasma diagnosis.

**Table 3:** Mental, physical, and social health in participants with melasma (n=746) stratified by race and ethnicity compared with age, sex, and income-matched controls (n=7460).

Comparison	Low Mental Health (aOR [95% CI], p-value)	Low Ability for Social Roles (aOR [95% CI], p-value)	Low Social Satisfaction (aOR [95% CI], p-value)	Low Physical Health (aOR [95% CI], p-value)
**Asian participants with vs. without melasma**	**4.04 [1.28, 12.8], p=0.02**	**3.46 [1.19, 10.1], p=0.02**	**3.07 [1.13, 8.3], p=0.03**	0.70 [0.22, 2.24], p=0.6
**White participants with vs. without melasma**	0.76 [0.50, 1.15], p=0.2	0.86 [0.55, 1.36], p=0.5	0.67 [0.44, 1.02], p=0.06	0.80 [0.57, 1.13], p=0.2
**Black participants with vs. without melasma**	0.73 [0.34, 1.56], p=0.4	0.82 [0.34, 1.76], p=0.6	0.75 [0.39, 1.43], p=0.4	0.69 [0.37, 1.27], p=0.2
**Other race participants with vs. without melasma**	1.19 [0.44, 3.23], p=0.7	1.19 [0.40, 3.53], p=0.8	1.92 [0.79, 4.67], p=0.2	0.40 [0.14, 1.14], p=0.09
**Hispanic or Latino participants with vs. without melasma**	1.04 [0.68, 1.59], p=0.8	1.16 [0.72, 1.86], p=0.6	1.21 [0.78, 1.87], p=0.4	0.91 [0.64, 1.31], p=0.6

aOR: Adjusted Odds Ratio

CI: Confidence Interval

**Table 4:** Mental, physical, and social health in participants with melasma and pre-existing mood disorders (n=230), anxiety disorders (n=206), stress disorders (n=130), substance use disorders (n=65), or sleep disorders (n=39) stratified by income compared to controls with the corresponding mental health disorders matched at a 1:10 ratio on age, sex, race/ethnicity, and income.

	Low Mental Health (aOR [95% CI], p-value)	Low Ability for Social Roles (aOR [95% CI], p-value)	Low Social Satisfaction (aOR [95% CI], p-value)	Low Physical Health (aOR [95% CI], p-value)
**Participants with prior mood disorders with vs. without melasma**	**1.32 [1.01, 1.72], p=0.04**	1.00 [0.74, 1.33], p=1.0	0.94 [0.72, 1.24], p=0.7	0.87 [0.68, 1.11], p=0.3
**Participants with prior anxiety disorders with vs. without melasma**	**1.38 [1.06, 1.81], p=0.02**	0.91 [0.68, 1.23], p=0.5	0.84 [0.64, 1.11], p=0.2	1.38 [1.07, 1.77], p=.01
**Participants with prior stress disorders with vs. without melasma**	0.89 [0.63, 1.27], p=0.5	1.27 [0.87, 1.85], p=0.2	0.97 [0.68, 1.40], p=0.9	1.01 [0.73, 1.41], p=0.9
**Participants with prior substance use disorders with vs. without melasma**	0.85 [0.50, 1.46], p=0.6	1.23 [0.69, 2.19], p=0.5	1.06 [0.62, 1.84], p=0.8	1.17 [0.71, 1.92], p=0.5
**Participants with prior sleep disorders with vs. without melasma**	0.83 [0.63, 1.10], p=0.2	1.00 [0.74, 1.34], p=1.0	0.91 [0.68, 1.20], p=0.5	0.77 [0.60, 0.99], p=0.04

aOR: Adjusted Odds Ratio

CI: Confidence Interval

**Table 5:** Mental, physical, and social health in participants with melasma (n=746) stratified by income compared with age, sex, and race-matched controls (n=7460).

Comparison	Low Mental Health (aOR [95% CI], p-value)	Low Ability for Social Roles (aOR [95% CI], p-value)	Low Social Satisfaction (aOR [95% CI], p-value)	Low Physical Health (aOR [95% CI], p-value)
**Income <$25k participants with vs. without melasma**	1.07 [0.72 – 1.60], p=0.7	0.80 [0.43 – 1.49], p=0.5	1.44 [0.57 – 3.65], p=0.4	1.30 [0.89 – 1.90], p=0.2
**Income $25k-50k participants with vs. without melasma**	0.82 [0.48 – 1.43], p=0.5	2.35 [0.74 – 7.44], p=0.2	1.21 [0.79 – 1.85], p=0.4	1.49 [0.98 – 2.26], p=0.1
**Income $50k-100k participants with vs. without melasma**	0.71 [0.41 – 1.23], p=0.2	1.31 [0.89 – 1.95], p=0.2	1.04 [0.73 – 1.50], p=0.8	0.68 [0.42 – 1.06], p=0.09
**Income $100k-200k participants with vs. without melasma**	1.70 [0.62 – 4.68], p=0.3	1.01 [0.62 – 1.63], p=1.0	1.29 [0.61 – 2.74], p=0.5	1.03 [0.74 – 1.42], p=0.8
**Income over $200k participants with vs. without Melasma**	1.01 [0.67 – 1.52], p=1.0	**0.51 [0.31 – 0.84], p=0:01**	1.07 [0.73 – 1.59], p=0.6	1.05 [0.71 – 1.55], p=0.8

aOR: Adjusted Odds Ratio CI: Confidence Interval

## Data Availability

This study used the *All of Us* Research Program’s Controlled Tier Dataset version 7, available to authorized users on the Researcher Workbench.
